# Flowering Phenology and the Influence of Seasonality in Flower Conspicuousness for Bees

**DOI:** 10.3389/fpls.2020.594538

**Published:** 2021-02-16

**Authors:** Amanda Eburneo Martins, Maria Gabriela Gutierrez Camargo, Leonor Patricia Cerdeira Morellato

**Affiliations:** Phenology Laboratory, Department of Biodiversity, Biosciences Institute, São Paulo State University (UNESP), Rio Claro, Brazil

**Keywords:** background coloration, bee visual system, cerrado *sensu stricto*, color contrasts, flower color diversity, flowering patterns, plant–animal communication, plant community

## Abstract

Flowering patterns are crucial to understand the dynamics of plant reproduction and resource availability for pollinators. Seasonal climate constrains flower and leaf phenology, where leaf and flower colors likely differ between seasons. Color is the main floral trait attracting pollinators; however, seasonal changes in the leaf-background coloration affect the perception of flower color contrasts by pollinators. For a seasonally dry woody cerrado community (Brazilian savanna) mainly pollinated by bees, we verified whether seasonality affects flower color diversity over time and if flower color contrasts of bee-pollinated species differ between seasons due to changes in the leaf-background coloration. For 140 species, we classified flower colors based on human-color vision, and for 99 species, we classified flower colors based on bee-color vision (spectral measurements). We described the community’s flowering pattern according to the flower colors using a unique 11 years phenological database. For the 43 bee-pollinated species in which reflectance data were also available, we compared flower color diversity and contrasts against the background between seasons, considering the background coloration of each season. Flowering was markedly seasonal, peaking at the end of the dry season (September), when the highest diversity of flower colors was observed. Yellow flowers were observed all year round, whereas white flowers were seasonal, peaking during the dry season, and pink flowers predominated in the wet season, peaking in March. Bee-bluegreen flowers peaked between September and October. Flowers from the wet and dry seasons were similarly conspicuous against their corresponding background. Regardless of flowering season, the yellowish background of the dry season promoted higher flower color contrast for all flower species, whereas the greener background of the wet season promoted a higher green contrast. Temporal patterns of flower colors and color contrasts were related to the cerrado seasonality, but also to bee’s activity, visual system, and behavior. Background coloration affected flower contrasts, favoring flower conspicuousness to bees according to the season. Thus, our results provide new insights regarding the temporal patterns of plant–pollinator interactions.

## Introduction

In tropical ecosystems, seasonal changes in rainfall, temperature, and day length are the primary flowering constraints (Morellato et al., 2000; Abrahamczyk et al., 2011; Cortés-Flores et al., 2017). Seasonality shapes flowering patterns, which are strongly related to the dynamics of plant communities, defining temporal changes in plant reproduction, resource availability for flower visitors, maintenance of plant–pollinator diversity, and plant reproductive success (Gentry, 1974; Lieth, 1974; Ramírez, 2006; Morellato et al., 2016). Thus, concentrating flowering in periods with favorable weather conditions could increase pollinators’ diversity and activity (Frankie et al., 1974; Newstrom et al., 1994; Ramírez, 2006). Additionally, flowering patterns are also shaped by biotic factors, mainly plant–pollinator interactions (Ramírez, 2006; Cortés-Flores et al., 2017). Pollinators exert a key selective pressure on flowering, affecting the intensity, productivity, and length of the reproductive season and also species’ synchronicity (Augspurger, 1981, 1983; Smith-Ramírez et al., 1998).

Flowers attract pollinators by distinct stimuli, such as color, shape, size, and scent (Fenster et al., 2004). However, color is the main floral trait related to pollinator attraction and plant–animal communication; flower color is adapted to pollinators’ visual sensitivity and preferences (Lunau and Maier, 1995; Chittka et al., 1999; Fenster et al., 2004; Dyer et al., 2006). Hence, the diversity of flower colors in angiosperms is mainly a consequence of the selective pressures exerted by pollinators (Chittka and Menzel, 1992; Dyer et al., 2012; Shrestha et al., 2013; Camargo et al., 2019). Bees are the dominant pollinators in several plant communities and biomes around the world, mainly due to their large morphological variation, distinct foraging behaviors, total dependency on floral resources, and highly developed communication system (Ollerton, 2017). Although Hymenoptera are active throughout the year, a higher activity, especially in bees, has been observed mainly between November and June in cerrado woodland (D’Avila and Marchini, 2008). Eusocial bees, such as *Apis mellifera* and *Trigona spinipes*, are less susceptible to temperature and humidity variation, being important pollinators during drier periods when less resources are available (D’Avila and Marchini, 2008).

Bees, as most Hymenoptera, possess a trichromatic color vision and perceive colors based on photoreceptors sensible to green, blue, and ultraviolet (UV) wavelengths (Chittka and Menzel, 1992). In general, bees prefer flowers that reflect short wavelengths and saturated colors (higher spectral purity), where the contrast against the background is a key signal for flower distinction and detection (Rohde et al., 2013; Telles et al., 2017; Camargo et al., 2019).

The background promoting flower contrast is composed mainly by leaves that change colors throughout the year in seasonal ecosystems (Alberton et al., 2014; Camargo et al., 2014). In tropical seasonally dry forests, most species are deciduous or semi-deciduous, losing their leaves in the dry season (Alberton et al., 2014, 2019; Camargo et al., 2014, 2018). During senescence, leaves lose chlorophyll, altering the background color from green (formed by young and mature leaves in the rainy season) to yellowish, brownish, or reddish in the dry season (Camargo et al., 2014). Thus, differences in the background color may affect the flower color contrast and, consequently, flower detection and discrimination by bees (Camargo et al., 2014; Binkenstein and Schaefer, 2015; Bukovac et al., 2017a; Telles et al., 2017).

Using a community-level approach and the cerrado as a model of a tropical seasonally dry vegetation, we investigated the seasonality of flowering phenology, the flower color diversity, and the respective flower color contrasts according to the visual system of bees. Specifically, we asked: (i) Does seasonality influence the diversity of flower colors over time? (ii) Do the color contrasts of bee-pollinated flowers differ between seasons according to changes in the background coloration? (iii) Are distinct bee-flower colors and contrasts over seasons enhancing flower conspicuousness? Flowers are observed throughout the year in the cerrado; however, flowering phenology is seasonal, peaking at the end of the dry season (Batalha and Martins, 2004; Gottsberger and Silberbauer-Gottsberger, 2006; pers. obs). Therefore, we expected the highest diversity of flower colors during the dry season, a period that favors flowering and bees’ activity (Morellato and Leitão-Filho, 1996; Gottsberger and Silberbauer-Gottsberger, 2006). We also expected that bee-flower colors and its associated visual contrasts shift between seasons to increase flower contrast against the background of the corresponding flowering season.

## Materials and Methods

### Study Area

The Brazilian cerrado is a Neotropical savanna that contains different vegetation types, from grasslands to woodlands, with high ecological and landscape diversity under a seasonally dry climate (Klink and Machado, 2005; Coutinho, 2006).

Our study was carried out in a cerrado woodland located in Itirapina, São Paulo State, southeastern Brazil (22°10′31.41?S, 47°52′26.13″W). The vegetation of the study area is mainly the cerrado *sensu stricto* (Reys et al., 2013), hereafter called cerrado, which is dominated by woody species and is the typical and most widespread savanna physiognomy of the Brazilian cerrado (Coutinho, 2006; Supplementary Figures S1A,B). The vegetation is classified as semi-deciduous and is characterized by a continuous herbaceous layer, scattered shrubs, and a discontinuous tree cover that reaches from 6 to 12 m high (Reys et al., 2013; Camargo et al., 2018; Supplementary Figure S1C). In the cerrado, flowering is seasonal, and most woody species are pollinated by bees (Oliveira and Gibbs, 2002; Gottsberger and Silberbauer-Gottsberger, 2006). The seasonal climate is marked by a rainy warm season from October to March and a dry and cooler season from April to September (Camargo et al., 2018). The mean annual temperature is 20°C, and the annual total rainfall is 1,524 mm (Camargo et al., 2018). The studied cerrado community shows a high diversity of shrubs and trees, with the most species-rich families being Myrtaceae, Fabaceae, Malpighiaceae, and Vochysiaceae and the most abundant species being *Bauhinia rufa* (Bong.) Steudel, *Xylopia aromatica* (Lam.) Mart., *Miconia rubiginosa* (Bonpl.) A.D., *Virola sebifera* (Aubl), and *Myrcia guianensis* (Aubl.) DC. (Reys et al., 2013).

### Plant Survey and Phenology Monitoring

The studied species were selected from a list of 222 woody plant species sampled during a floristic survey carried out every month at the study site since 2004 (Reys et al., 2013). The systematic plant survey was conducted in 36 25 × 2 m transects 50 m apart from each other, where all woody plants with a circumference above the ground >3 cm were tagged, sampled, identified to species level, and monitored individually for reproductive and vegetative phenology (Reys et al., 2013; Camargo et al., 2018; Escobar et al., 2018).

We collected flower reflectance data at least once a month from April 2017 to April 2018, for which we searched and sampled flowering species throughout the study area. We analyzed 140 animal-pollinated plant species previously surveyed (see above) belonging to 97 genera and 44 families (Supplementary Table S1). The most representative families were Fabaceae (15%), Bignoniaceae (8.5%), Asteraceae (8%), Malpighiaceae (7%), and Myrtaceae (6%). From the 140 animal-pollinated plant species, 91 were monitored for phenology, and 99 were measured for flower reflectance. However, we only have phenology and spectral measurements for 49 species. We inferred the pollinators for the 140 plant species compiled and found that 115 plant species have bees as the main or secondary pollinator (Supplementary Table S1). The pollinators of the studied species were extracted from an extensive review for cerrado pollinators performed by Martins (2019). Vouchers of the plant species sampled are lodged in the Herbarium Rioclarense (HRCB) of the São Paulo State University (UNESP).

### Flowering Phenology

Phenological observations have been carried out once a month in the 36 plant systematic survey transects since September 2004 by the Phenology Laboratory team (UNESP—São Paulo State University, campus Rio Claro) as part of the cerrado long-term phenology monitoring program (for details, see Camargo et al., 2013, 2018; Vogado et al., 2016; Escobar et al., 2018). For each observed individual, vegetative (leaf flush and leaf fall) and reproductive (flower buds, anthesis, and unripe and ripe fruits) phenophases are scored using a semi-quantitative index represented by the given classes of intensity: 0 (absence), 1 (≤50% of branches active, intermediate intensity), and 2 (>50% of branches active, peak of intensity) (Opler et al., 1980; Vogado et al., 2016). From the phenology monitoring database, we selected all species that flourished at any given year from January 2005 to December 2015. Hence, we evaluated the temporal patterns of flower color diversity in the cerrado community and related species flowering time to the respective flower color according to human- and bee-color categories, similar to Camargo et al. (2013) in which fruiting patterns were analyzed according to fruit color in the same cerrado area.

### Flower Colors and Contrasts

We classified species flower colors according to the human visual system as white (including whitish), yellow, pink (including violet and blue flowers), green, and red (including brown and orange). When available, we used the reflectance spectra to determine the human color using the function *spec2rgb* from the *Pavo* package (Maia et al., 2018) in R (R Development Core Team). For species in which we had the reflectance data, we also classified flower colors according to the visual systems of bees as described below. The low number of species in certain color categories is a consequence of the flower color frequency found for cerrado areas (Oliveira and Gibbs, 2000, 2002; Gottsberger and Silberbauer-Gottsberger, 2006).

We measured the flower spectral reflectance between 300 and 700 nm, including the UV light, using a spectrophotometer (Ocean Optics—Jaz Modular Optical Sensing Suite) equipped with a pulsed xenon light source with a spectral range between 190 and 1,100 nm. The pulsed xenon light avoids the possible degradation of samples by the UV light. To calibrate the equipment and record the reflectance data, we used a PTFE disc (WS-1 Diffuse Reflectance Standard, PTFE—Ocean Optics) as the white standard and a black suede paper as the black standard. Flowers were preserved in individual bags stored in cooler boxes until the measurements were carried out, in the laboratory, on the same day. Flower color was based on the mean reflectance spectra calculated from the reflectance data of 10 flowers (collected from different individuals) for each species (Dalrymple et al., 2015). Flower color was considered as the reflectance spectra of the predominant color in the floral display, which generally corresponded to the color of the petal (Camargo et al., 2019).

To evaluate flower colors and calculate color variables according to the visual systems of bees, we used the bee hexagon proposed by Chittka (1992), which represents the bee-color space. Each photoreceptor present on the retina of the bees’ eye (UV, blue, and green) is represented in a vertex of the bee hexagon. Each flower color is represented by a point (color loci) in the hexagon that corresponds to the Euclidian distance between the flower background and the stimulus promoted by the light reflected from the flower in each photoreceptor, according to a previously specified visual system (Chittka, 1992). To calculate the flower color loci in the bee hexagon, we used the visual model of *A. mellifera* proposed by Menzel and Backhaus (1991), the D65 standard daylight, and the reflectance of leaves collected from the plant community in the dry and wet seasons based on Camargo et al. (2013). The leaf background of each season is represented by the mean reflectance spectra composed by leaves of different species collected in November (wet season), when leaves are completely developed after the peak of leaf flushing in September, and in July (dry season) when the peak of leaf fall occurs (Camargo et al., 2013, 2014, 2018). According to the color loci position in the bee hexagon, we classified flower colors in six bee-color categories: bee-blue, bee-green, bee-UV, bee-bluegreen, bee-UVgreen, and bee-UVblue (Chittka et al., 1994).

To analyze differences in the color contrasts between bee-pollinated flowers of the dry and wet seasons, we calculated the color and green contrasts promoted by flowers produced in each season. For such, we calculated the contrasts promoted by flowers produced mainly in the wet season (flowering peak between October and March) against the background of the wet season and calculated the contrasts promoted by flowers produced during the dry season (flowering peak between April and September) against the background of the dry season. We also calculated and compared the contrasts of flowers produced in the dry and wet seasons against the background of their opposite season. The color contrast or chromatic contrast is the *r* vector in the bee hexagon that is represented by the Euclidian distance between a given flower color loci and the hexagon center, corresponding to the background color locus (Chittka, 1992). The color contrast is important for bees to detect flowers from the background and is activated only at short distances and large visual angles (Spaethe et al., 2001; van der Kooi et al., 2019). The green contrast or achromatic contrast is the contrast between two colors detected by the green photoreceptor and adapted to the background; it is used for long-distance detection and is always active when bees are foraging (Chittka, 1992; Spaethe et al., 2001; van der Kooi et al., 2019). The green contrast against the background corresponds to the green photoreceptor excitation adjusted to the background, that is, the green photoreceptor stimulus subtracted by 0.5 (Spaethe et al., 2001; Dyer et al., 2016).

### Data Analyses

We analyzed the flowering phenology using the flower peak date of each species, defined as the most recurrent month with maximum flowering intensity of a given species throughout the 11 years of observations (see Escobar et al., 2018). To test for seasonality in the flowering peak of the community and for each flower color, we used circular statistics based on Morellato et al. (2000, 2010). For such, the flower peak date (month of the year) of each species was converted to an angle or vector direction (15°corresponds to January and so on), after which we calculated the mean angle and angular standard deviation (Morellato et al., 2010) for the community and for each flower color. We then applied the Rayleigh test (Z) to test for the significance of the mean angle or mean date, i.e., whether the species’ flower peak dates or angles are significantly concentrated around the mean angle or date (Morellato et al., 2010). If the mean angle is significant, the flower peak pattern is considered seasonal, and the degree of seasonality (of the community or for each flower color) is measured as the length of the mean vector (*r*): the *r* vector ranges from 0, no concentration or no seasonality, to 1, highest concentration around the mean angle or highest degree of seasonality (Morellato et al., 2000, 2010).

We also calculated the linear flowering pattern based on an intensity index calculated by a modified Fournier index (Fournier, 1974) that uses two instead of four classes of intensity (Vogado et al., 2016). This index was calculated for each species as the sum of the intensity classes of the individuals in a given month divided by the total number of individuals of this species multiplied by two, the maximum intensity class (Vogado et al., 2016). For each species, we calculated a unique mean year to represent the flowering pattern using the intensity indices calculated for 11 years. We then calculated the flowering pattern based on this index for the community (all species), for bee-pollinated species, and for species grouped by flower colors according to the human vision.

To compare the color contrasts of bee-pollinated flowers produced mainly during the dry (April to September) and wet (October to March) seasons, we applied a Wilcoxon rank sum test (W) with a continuity correction to compare the quantitative variables of color and green contrasts between the seasons. To verify if seasonal changes in the background coloration interfere with flower conspicuousness, we compared the flower contrasts against the background of the corresponding flowering season and against the background of the opposite season using the Wilcoxon paired signed rank test (V).

Circular statistics were carried out in ORIANA 4.0 (Kovach, 2011) and linear statistics in R (R Development Core Team). In addition, we analyzed the reflectance spectra and calculated the color variables using the *Pavo* package in R (Maia et al., 2018).

## Results

### Cerrado Plant Pollinators and Flower Colors

Bees were the dominant pollinators: 74% of the 140 plant species were mainly pollinated by bees or 82% if we added plant species that have bees as secondary pollinators (Supplementary Table S1).

**FIGURE 1:**
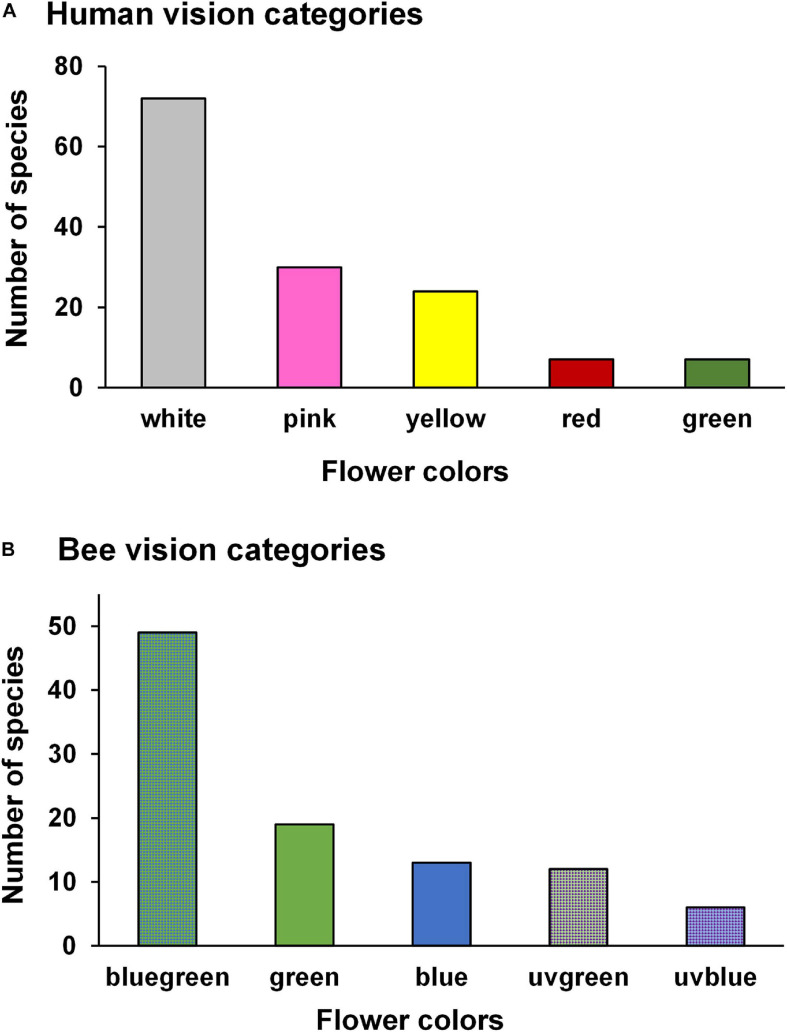
Number of species in each flower color according to the human- **(A)** and bee- **(B)** color vision for 140 and 99 species, respectively, sampled in a woody cerrado. **(A,B)** Include species with different pollinators.

According to the human eye, white and pink were the most frequent flower colors (52 and 21%, respectively), followed by yellow (17%), green (5%), and red (5%) (Figure 1A). Among the 99 species with reflectance data, bee-bluegreen represented half of the species (50%), followed by bee-green (18%), bee-blue (13%), bee-UVgreen (12%), and bee-UVblue (6%) (Figure 1B).

### Flowering, Color Patterns, Pollination, and Seasonality

**FIGURE 2:**
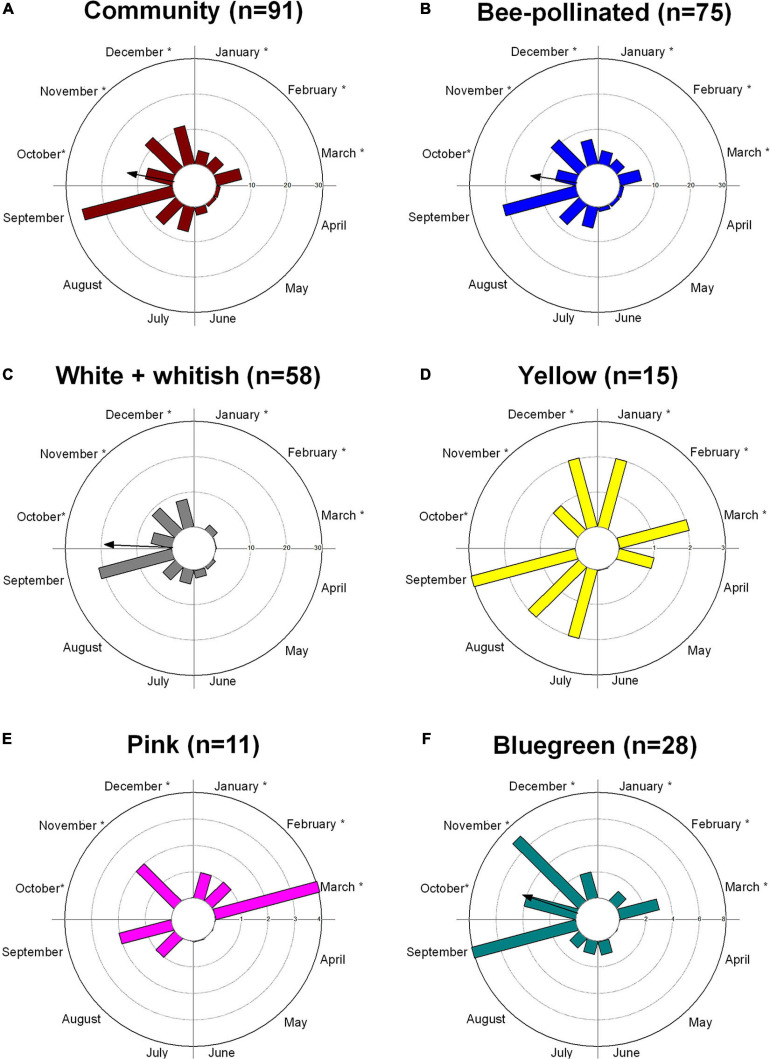
Flowering patterns of a woody cerrado vegetation (Itirapina, southeastern Brazil) based on the number of species presenting flowering peak in each month: **(A)** for the cerrado community (91 species), **(B)** for 75 species pollinated by bees, **(C–E)** by flower color according to the human-color vision, and **(F)** for the bee-bluegreen flowers, the predominant bee color in the community. The arrows point to the significant mean angles or dates, and the arrows’ length corresponds to the *r* vector value (0–1). Arrows are not presented for bimodal patterns **(D,E)**. The rainy warm season occurs from October to March, represented by an *, and the dry cooler season from April to September; a transitional dry-to-wet season is observed between September and October.

Flowering was observed throughout the year in the community based on data of 91 woody animal-pollinated species included in the phenology monitoring. The community flowering peak occurred in September, at the end of the dry season (Figure 2A and Supplementary Figure S2A), which showed the highest diversity of flowers available for pollinators (29% of species), followed by December (14%) and November (10.5%). From the 91 species analyzed, 83% (75 species) were bee-pollinated (Supplementary Table S1). Bee-pollinated flowers were available all year long, but showed a higher availability in September (22 species) and November (13 species) and a lower availability in April, May, and June (only 1 species in each month) (Figure 2B and Supplementary Figure S2B). The flowering pattern was significantly seasonal (Z = 14.7; *p* = 0.001; vector *r* = 0.44) with a mean date in October (Figure 2B). For the dataset of 49 species with phenological and reflectance data, the flowering pattern was also significantly seasonal (Z = 13.6; *p* = 0.001; vector *r* = 0.52), and the mean date was again in October, peaking in September (Supplementary Figure S3).

Flowering phenology showed that white flowers (according to human colors; *n* = 58) presented a significant seasonal pattern, peaking in September (Figure 2C and Supplementary Figure S2C) with a mean date in October (Z = 13.29; *p* < 0.001; vector *r* = 0.60). On the other hand, yellow flowers (*n* = 15) were more distributed along the year, where no peak and no significant seasonality (Z = 0.5; *p* = 0.6) were observed (Figure 2D and Supplementary Figure S2D). Pink flowers bloomed mainly in the wet season, peaking at the end of the wet season (Figure 2E and Supplementary Figure S2E) but with no significant seasonality (Z = 0.8; *p* = 0.4). Bees pollinate 86% of white flowers, 90% of pink flowers, and 93% of yellow flowers.

Flowering of bee-bluegreen flowers, the predominant bee color in our community (28 species), was also significantly seasonal, peaking mainly at the end of the dry season (September) with a mean date in October (Z = 8.1; *p* = 0.001; vector *r* = 0.53) (Figure 2F). The other bee-color species were not contemplated in our phenological database.

### Flower Color and Contrasts in the Dry and Wet Seasons

**FIGURE 3:**
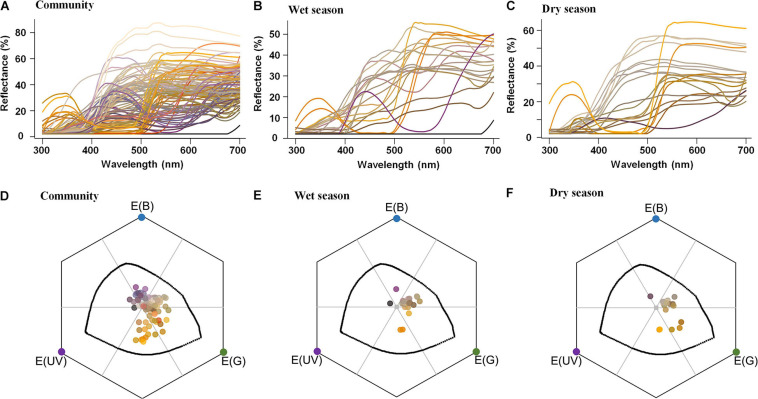
Reflectance spectra **(A–C)** and color loci distribution **(D–F)** in the bee visual space of flowers from 99 animal-pollinated cerrado species **(A,D)** and of flowers from 22 bee-pollinated species flowering mainly during the wet season **(B,E)** and 21 bee-pollinated species flowering mainly during the dry season **(C,F)**. Each line in the reflectance spectra **(A–C)** and each point in the hexagons **(D–F)** are represented by the correspondent human color according to the function *spec2rgb* for R (Maia et al., 2018). In the bee visual space, represented by hexagons **(D–F)**, the gray point represents the achromatic center (color locus of the leaf background), and the colorful points in the vertices represent each bee-photoreceptor: blue E (B), green E (G), and ultraviolet E (UV). The black line inside the hexagon delimits the maximum sensitivity of each photoreceptor to a monochromatic light. Each of the six parts of the hexagon, limited by a gray line, represents a bee-color category—clockwise direction from E (B): blue, bluegreen, green, UVgreen, UV, and UVblue.

From the 43 bee-pollinated species for which we had phenological and reflectance data, 22 showed a flowering peak during the rainy season (from October to March; Figures 3B,E), whereas 21 peaked during the dry season (from April to September; Figures 3C,F). The percentage of bee-bluegreen flowers was similar between seasons: 67% of species with bee-bluegreen flowers peaked in the dry season and 64% in the wet season (Figure 3 and Supplementary Table S1). Conversely, the percentage of species with bee-green flowers peaking in the dry season was higher (14%) than that in the wet season (4.5%), whereas 18% of species with bee-blue flowers peaked in the wet season, and only 5% (one species) in the dry season (5%) (Figure 3 and Supplementary Table S1).

**FIGURE 4:**
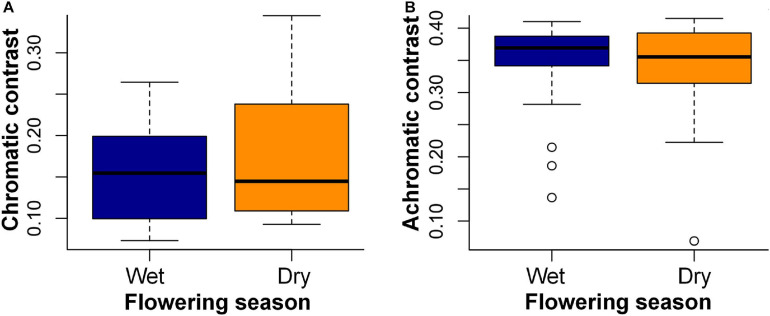
Main flowering season and flower contrasts against the leaf background calculated for 43 bee-pollinated species sampled in the study site. **(A)** Color contrast and **(B)** green contrast against the leaf background of the dry and wet seasons. Horizontal lines indicate the mean value of contrasts, and vertical lines indicate the standard deviation. Chromatic contrasts are given in hexagon units and correspond to the Euclidian distance between a flower color locus and the center of the bee hexagon; the green contrasts are stimulus of the green photoreceptor adapted to the background (Chittka, 1992).

Floral contrasts did not differ between seasons: color contrast (W = 212, *p* = 0.65) and green contrast (W = 235, *p* = 0.93) (Figures 4A,B). Additionally, flowers of both seasons showed higher values of color contrast against the background during the dry season (dry season flowers: V = 225, *p* < 0.001; wet season flowers: V = 6, *p* < 0.001) and higher values of green contrast against the background during the wet season (dry season flowers: V = 0, *p* < 0.001; wet season flowers: V = 232, *p* < 0.001).

## Discussion

The plant species in the cerrado community studied flowered throughout the year, where the highest diversity of flowering species and flower colors was observed in September, at the end of the dry season. Bee pollination was the dominant pollination system with bee-pollinated flowers available all year long, decreasing in quantity only during the early dry season (April to June). White and bee-bluegreen are the most common flower colors according to human and bee visual systems, respectively. Flowers produced during the dry and wet seasons showed similar contrasts against their corresponding background. However, regardless of the flowering season, the background of the dry season promoted the highest color contrasts, whereas the green contrast was higher against the wet season background.

### Bees and Flower Color Patterns

Based on the human visual system, white, yellow, and pink were the most common flower colors, similar to what has been described for other cerrado areas (Oliveira and Gibbs, 2000, 2002; Gottsberger and Silberbauer-Gottsberger, 2006). Similarly, bees were responsible for the pollination of more than 70% of species in our community and at least 50% in other cerrado communities (Oliveira and Gibbs, 2000, 2002; Gottsberger and Silberbauer-Gottsberger, 2006). September was the month with the highest flowering intensity coupled with the highest occurrence of bees (D’Avila and Marchini, 2008). Bee activity coincides to the number of flowering species and resource availability: a greater number of bee species have been observed from August to September and from November to June (Gottsberger and Silberbauer-Gottsberger, 2006; D’Avila and Marchini, 2008).

The high flowering intensity and the high number of species reaching the flowering peak at the end of the dry season corroborate the pattern observed for other cerrado communities (Oliveira and Gibbs, 2000). Even though precipitation, temperature, and photoperiod are triggers that induce flowering of cerrado species (Batalha et al., 1997; Oliveira, 2008; Pirani et al., 2009), cerrado woody species are less dependent on seasonal restrictions, and water availability is not a limiting factor for flowering (Batalha et al., 1997; Pirani et al., 2009). Moreover, resource allocation, pollinator competition, phylogenetic restrictions, optimal dispersion period, and occasional fire events are also important factors that can affect flowering (Miranda et al., 1998; Oliveira, 2008; Escobar et al., 2018).

Flowers of all colors peaked at the end of the seasons, considered a transition between seasons (Escobar et al., 2018). The predominant white and yellow flowers peaked at the end of the dry season, whereas pink flowers peaked at the end of the wet season. The pink flower pattern may be related to the predominance of wind dispersal among these species, in which seeds must be dispersed during the dry season (Batalha and Martins, 2004; Escobar et al., 2018). In seasonal forests, the dry season has long been hypothesized to favor pollination by insects due to high light availability and low precipitation (Janzen, 1967). In the cerrado, eusocial Apidae bees are less susceptible to low temperatures and low relative humidity and are considered active pollinators of cerrado plant species all year long (Almeida and Laroca, 1988; D’Avila and Marchini, 2008; Abrahamczyk et al., 2011). Considering the importance of *A. mellifera* and *T. spinipes* as pollinators in cerrado communities (D’Avila and Marchini, 2008), yellow and white flowers are definitely important food sources during the dry season, maintaining pollinator activity all year long (Ramírez, 2006). In addition, by flowering during the dry season, these species avoid competition, decreasing the interference of other flowering plants (Ramírez, 2006).

Species with bee-bluegreen flowers peaked mainly at the end of the dry season (September), but flowering was observed throughout most of the year, confirming the importance of these flowers as food sources for different pollinators. According to the trichromatic vision of bees, bee-bluegreen was the most common flower color in the cerrado. This bee color includes the bee-pollinated flowers perceived by humans as white or whitish (Kevan et al., 1996), corroborating the importance of bees as pollinators in the cerrado. In addition, the bee-bluegreen color also includes pale colors related to many other groups of pollinators, such as moths, flies, beetles, and bats (Gottsberger and Silberbauer-Gottsberger, 2006). Even though information regarding UV reflectance is lacking, we know, for example, that the bee-UVgreen and bee-green flowers correspond to human-yellow flowers (Kevan et al., 1996), which were produced throughout the year, and that the bee-blue and bee-UVblue flowers correspond mainly to the human-pink flowers (Kevan et al., 1996), which were more concentrated in the wet season.

### Seasonality and Visual Contrasts

Flower color contrasts were affected by abiotic conditions as the background coloration was significantly different between the dry and wet seasons (Camargo et al., 2014), suggesting that it acts as a selective pressure for flower color and detection. Foraging in complex backgrounds are challenging to bees, as fluctuations in the perceived contrasts are frequent when compared with a homogeneous achromatic background (Telles et al., 2017).

Even though flower color phenology differed, flower contrasts were similar between seasons. Plants flowering in the wet and dry seasons were equally conspicuous against their corresponding background. However, we found that the background coloration affected flower contrasts regardless of flowering season. The leaf senescence peak in our community occurs in the dry season, promoting a yellowish background (Alberton et al., 2014; Camargo et al., 2018) and thus higher color contrast, which most likely maximizes flower detection in a period under less favorable environmental conditions and of reduced flower activity (Bukovac et al., 2017b; Telles et al., 2017). Most cerrado plants produce new leaves at the end of the dry season, with a predominance of greener leaves throughout the wet season (Alberton et al., 2014; Camargo et al., 2018). The higher green contrast against the background of the wet season may be important to guarantee flower detection against new and mature green leaves. Hence, the different color contrasts are favored according to their importance for flower detection, ensuring flower conspicuousness across seasons. It is worth highlighting that during the transitions between seasons, the background coloration is also changing and can vary from a more yellowish to green (or greener to yellow) according to variations in the length of the seasons and associated leafing patterns over the years (Alberton et al., 2014; Camargo et al., 2018). Therefore, in seasonally dry ecosystems, where flowering peaks mainly at the end of the seasons (Morellato et al., 2013), it is also advantageous to produce flowers that contrast with the leaf coloration of both seasons.

We evaluated 11 years of phenology data to show, for the first time, that seasonality affects flower color diversity and flower contrasts in seasonal ecosystems, such as the cerrado. Distinctions in the peak of certain flower colors along with the maintenance of color diversity over the year may favor the presence and diversity of bees and other pollinators throughout the year in the community (Ramírez, 2006; Genini, 2011). We have demonstrated the importance of considering the bee-color vision associated to the natural background and its seasonal changes when analyzing flower color contrasts. Background coloration influenced color contrasts, favoring flower conspicuousness to bees according to seasons. When linking flower color, seasonality, and bee pollination, we found that temporal patterns of flower colors are likely adapted to abiotic and biotic constraints, such as climate seasonality, bee activity, visual system, and behavior. Thus, our results provide new insights for future research regarding the temporal patterns of plant–pollination interactions in seasonally dry ecosystems.

## Data Availability Statement

The raw data supporting the conclusions of this article will be made available by the authors, without undue reservation, to any qualified researcher.

## Author Contributions

AEM, MGGC and LPCM conceived and designed the study. MGGC and AEM conducted the field work and analyzed the data. AEM wrote the original draft of the manuscript. LPCM provided financial support and management. All authors contributed to revisions and editions.

## Conflict of Interest

The authors declare that the research was conducted in the absence of any commercial or financial relationships that could be construed as a potential conflict of interest.

## Supplementary Material

Supplementary Figure 1 **(A,B)** Location of the cerrado study site at Itirapina, São Paulo State, Brazil (above with a yellow pin) and the study site in detail (below), where the four red lines, along which the 36 transects were distributed, represent the general location of plots where phenology species were sampled and are observed in the long-term cerrado phenology monitoring [map data: **(A)** Google, Image Landsat/Copernicus; **(B)** Google, Maxar Technologies]; **(C)** general view of the cerrado *sensu stricto* vegetation (photo: AEM).

Supplementary Figure 2 Flowering patterns of a woody cerrado vegetation (Itirapina, southeastern Brazil) based on the average monthly intensity. **(A)** For the cerrado community (91 species), **(B)** for 75 species pollinated by bees, **(C–E)** by flower color according to the human-color vision. The rainy warm season occurs from October to March, represented by an ^∗^, and the dry cooler season from April to September; a transitional dry-to-wet season is observed between September and October.

Supplementary Figure 3 Flowering patterns of a woody cerrado vegetation (Itirapina, southeastern Brazil) based on the number of species presenting flowering peak in each month, for 49 species with phenological and reflectance data. The rainy warm season occurs from October to March, represented by an ^∗^, and the dry cooler season from April to September; a transitional dry-to-wet season is observed between September and October.
