# Supplementary figures and images for: Flowering Phenology and the Influence of Seasonality in Flower Conspicuousness for Bees
Source: Front Plant Sci. 2021 Feb 16;11:594538. doi: 10.3389/fpls.2020.594538 (PMC7921784; doi:10.3389/fpls.2020.594538)

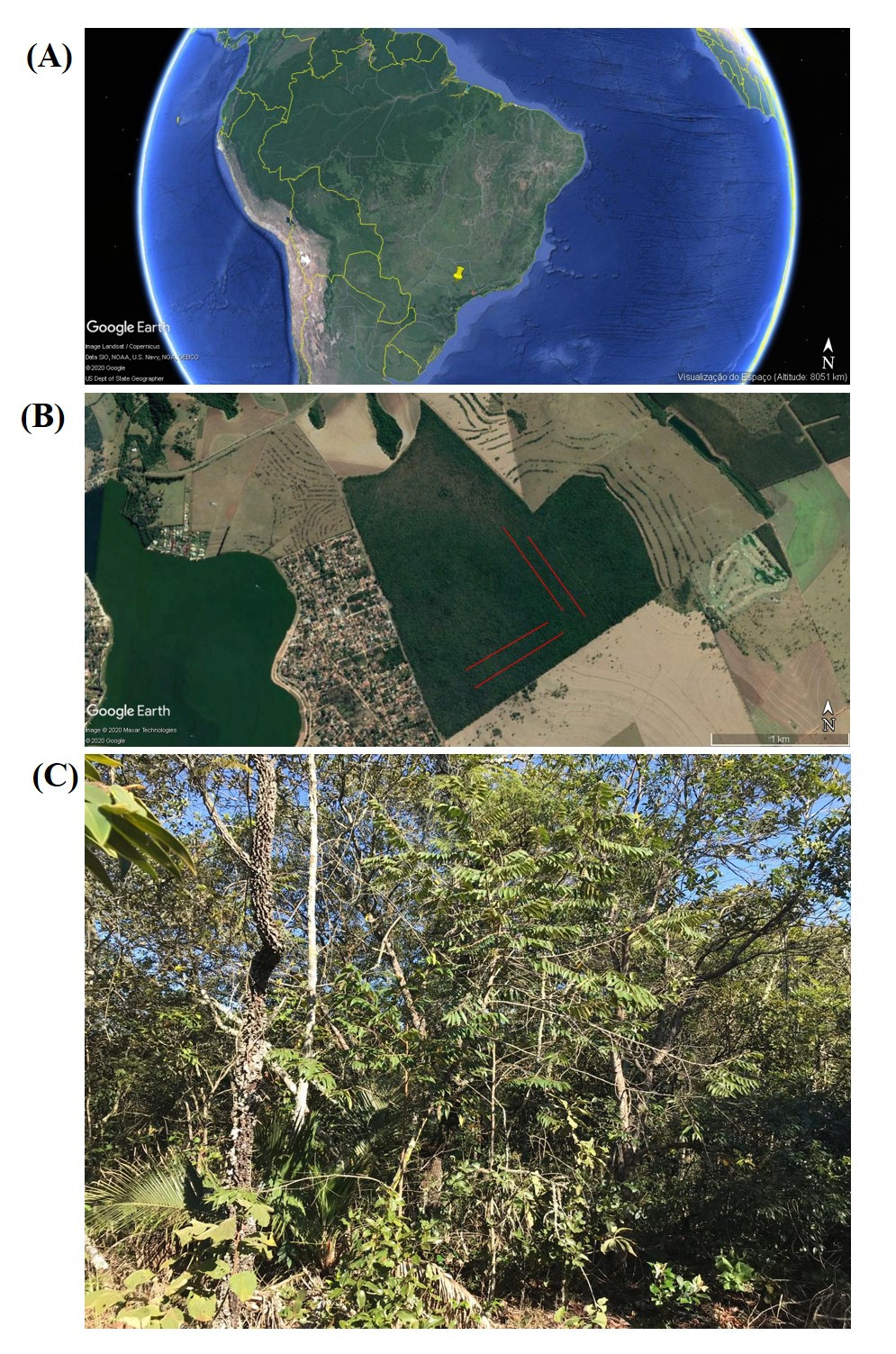

Supplement: Supplementary Figure 1 — (A,B) Location of the cerrado study site at Itirapina, São Paulo State, Brazil (above with a yellow pin) and the study site in detail (below), where the four red lines, along which the 36 transects were distributed, represent the general location of plots where phenology species were sampled and are observed in the long-term cerrado phenology monitoring [map data: (A) Google, Image Landsat/Copernicus; (B) Google, Maxar Technologies]; (C) general view of the cerrado sensu stricto vegetation (photo: AEM). [file Image_1.JPEG]

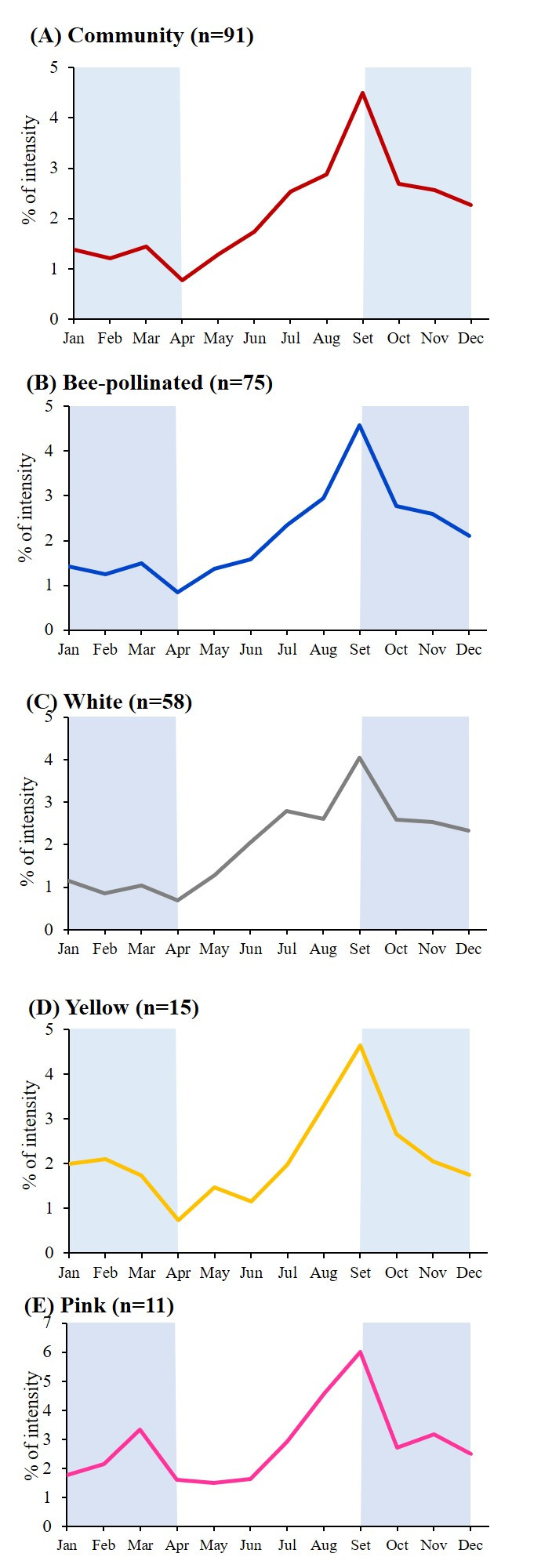

Supplement: Supplementary Figure 2 — Flowering patterns of a woody cerrado vegetation (Itirapina, southeastern Brazil) based on the average monthly intensity. (A) For the cerrado community (91 species), (B) for 75 species pollinated by bees, (C–E) by flower color according to the human-color vision. The rainy warm season occurs from October to March, represented by an ∗, and the dry cooler season from April to September; a transitional dry-to-wet season is observed between September and October. [file Image_2.JPEG]

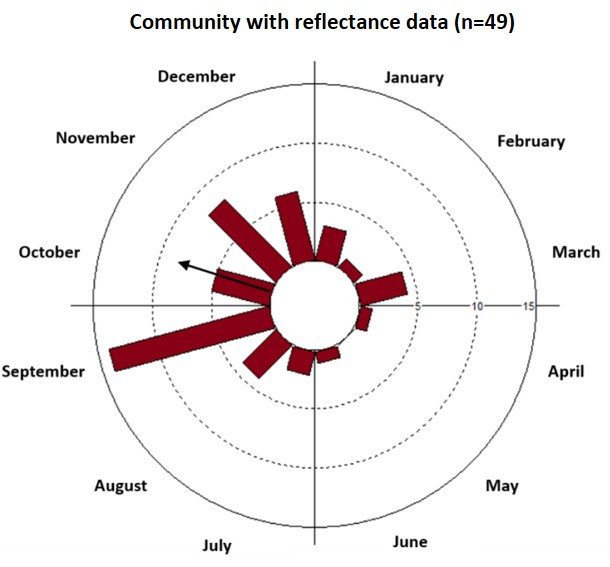

Supplement: Supplementary Figure 3 — Flowering patterns of a woody cerrado vegetation (Itirapina, southeastern Brazil) based on the number of species presenting flowering peak in each month, for 49 species with phenological and reflectance data. The rainy warm season occurs from October to March, represented by an ∗, and the dry cooler season from April to September; a transitional dry-to-wet season is observed between September and October. [file Image_3.JPEG]
