# Supplementary material for: Flowering Phenology and the Influence of Seasonality in Flower Conspicuousness for Bees
Source: Front Plant Sci. 2021 Feb 16;11:594538. doi: 10.3389/fpls.2020.594538 (PMC7921784; doi:10.3389/fpls.2020.594538)
Supplement: Supplementary file 4 [file Data_Sheet_1.docx]

**Pollinators References List -Table S1**

1 Imperatriz-Fonseca, V. L., Alves-dos-santos, I., Santos-Filho, P. S., Engels, W., Ramalho, M., Wilms, W., et al. (2011). Checklist of bees and honey plants from São Paulo State, Brazil. Biota Neotrop.

2 Oliveira, P. E., Gibbs, P. E., and Barbosa, A. A. (2004). Moth pollination of woody species in the Cerrados of Central Brazil: A case of so much owed to so few? Plant Syst. Evol. 245, 41–54. doi:10.1007/s00606-003-0120-0.

3 Cornelia, L., Mac Hado, I., Stefan, C. P., Erbar, C., and Leins, P. (2004). Pollination biology of a Mandevilla species (Apocynaceae), characteristic of NE-Brazilian inselberg vegetation. Botanische Jahrbücher für Systematik, Pflanzengeschichte und Pflanzengeographie 229-243.

4 Franco, A. L. M. (1991). Biologia floral de duas especies sincronopatricas de Prestonia (Apocynaceae). [dissertation/master’s thesis] [Campinas, SP]: University of Campinas.

5 Avila Jr, R., Oliveira, R., Pinto, C. E., and Amorim, F. W. (2015). Sphingidae e Flores no Brasil – Panorama e Perspectivas de Uso de Polinizadores 143–152.

6 Gottsberger, G., and Silberbauer-Gottsberger, I. (2006). Life in the Cerrado: Pollination and seed dispersal (Vol. 2). Reta Verlag.

7 Silva, C. I., Araújo, G., and de Oliveira, P. E. A. M. (2012). Distribuição vertical dos sistemas de polinização bióticos em áreas de cerrado sentido restrito no triângulo Mineiro, MG, Brasil. Acta Bot. Brasilica 26, 748–760. doi:10.1590/S0102-33062012000400004.

8 Deus, F. F., Do Vale, V. S., Schiavini, I., and Oliveira, P. E. (2014). Diversity of reproductive ecological groups in semideciduous seasonal forests. Biosci. J. 30, 1885–1902.

9 Cavallin, E. K. S. (2014). Influência de fatores biológicos e históricos no tempo de descrição das espécies de Angiospermas do cerrado. [dissertation/master’s thesis] [Brasília, DF]: University of Brasilia.

10 Carstensen, D. Personal observation.

11 Gonçalves, R. B., Melo, G. A. R., and Aguiar, A. J. C. (2009). A assembléia de abelhas (Hymenoptera, Apidae) de uma área restrita de campos naturais do Parque Estadual de Vila Velha, Paraná e comparações com áreas de campos e cerrado. Pap. Avulsos Zool. 49, 163–181. doi:10.1590/s0031-10492009001400001.

12 Kinoshita, L. S., Torres, R. B., Forni-Martins, E. R., Spinelli, T., Yu, J. A., and Constâncio, S. S. (2006). Composição florística e síndromes de polinização e de dispersão da mata do Sítio São Francisco, Campinas, SP, Brasil. Acta Bot. Brasilica 20, 313–327. doi:10.1590/S0102-33062006000200007.

13 Berjano, R., Ortiz, P. L., Arista, M., and Talavera, S. (2009). Pollinators, flowering phenology and floral longevity in two Mediterranean Aristolochia species, with a review of flower visitor records for the genus. Plant Biol. 11, 6–16. doi:10.1111/j.1438-8677.2008.00131.x.

14 Scudeller, V. V., Vieira, M. F., and Carvalho-Okano, R. M. (2008). Distribuição espacial, fenologia da floração e síndrome floral de espécies de Bignonieae (Bignoniaceae). Rodriguésia 59, 297-307.

15 Agostini, K. and Sazima M. (2003). Resources of ornamental plants for bee on campus of the State University of Campinas, São Paulo, Brazil. Bragantia 62, 3.

16 Yanagizawa, Y. A. N. P., and Maimoni-Rodella, R. C. S. (2007). Floral visitors and reproductive strategies in five melittophilous species of Bignoniaceae in Southeastern Brazil. Brazilian Arch. Biol. Technol. 50, 1043–1050. doi:10.1590/S1516-89132007000700015.

17 Barros, M. G. (2001). Pollination ecology of Tabebuia aurea (Manso) Benth. & Hook. and T. ochracea (Cham.) Standl. (Bignoniaceae) in Central Brazil cerrado vegetation. Rev. Bras. Botânica 24, 255–261.

18 Silva, C. I. (2009). Distribution on space and time of floral resources used by Xylocopa spp. and their interactions with plants of cerrado (stricto sensu) in the Triângulo Mineiro. [doctoral thesis] [Uberlândia, MG]: Federal University of Uberlândia.

19 Martins, F. Q., and Batalha, M. A. (2006). Pollination systems and floral traits in cerrado woody species of the upper taquari region (central Brazil). Brazilian J. Biol. 66, 543–552. doi:10.1590/S1519-69842006000300021.

20 Borges, H. B. N. (2000). Biologia reprodutiva e conservação do estrato lenhoso numa comunidade do cerrado. [doctoral thesis] [Campinas, SP]: University of Campinas.

21 Barros, M. G. (1998). Sistemas reprodutivos e polinização em espécies simpátricas de Erythroxylum P. Br. (Erythroxylaceae) do Brasil. Rev. bras. Bot. 21, 2.

22 Gottsberger, G., and Silberbauer-Gottsberger, I. (1988). Evolution of Flower Structures and Pollination in Neotropical Cassiinae (Caesalpiniaceae) Species. Phyt. 28, 293–320.

23 Almeida, N. M. (2014). Enantiostilia e relações reprodutivas em espécies da subtribo Cassiinae (Fabaceae - Caesalpinioideae). [doctoral thesis] [Recife, PE]: Federal Rural University of Pernambuco.

24 Freitas, C. V., and Oliveira, P. E. (2002). Reproductive biology of Copaifera langsdorffii Desf. (Leguminosae, Caesalpinioideae). Rev. Bras. Botânica 25, 311–321. doi:10.1590/s0100-84042002000300007.

25 Gibbs, P. and Sassaki, R. (1998). Reproductive biology of Dalbergia miscolobium Benth. (Leguminosae-papilionoidae) in SE Brazil: the effects of pistillate sorting on fruit-set. Annals of Botany 81,6.

26 Tavares, W. S., de Azevedo Pereira, A. I., de Sousa Freitas, S., Serrão, J. E., and Zanuncio, J. C. (2014). The chemical exploration of Dimorphandra mollis (Fabaceae) in Brazil, with emphasis on insecticidal response: A review. J. Sci. Ind. Res. (India) 73, 465–468.

27 Carvalho, P. E. R. (2014). Espécies arbóreas brasileiras. Embrapa Informação Tecnológica; Colombo, PR.

28 Lima, L. C. L., Silva, F. H. M., and Dos Santos, F. D. A. R. (2008). Palinologia de espécies de Mimosa L. (Leguminosae - Mimosoideae) do Semi-Árido brasileiro. Acta Bot. Brasilica 22, 794–805. doi:10.1590/S0102-33062008000300016.

29 Rocha, D. M. S. (2006). Aspectos taxonômicos, genéticos e reprodutivos de Pterodon pubescens (Benth.) Benth. E P. emarginatus Vog. (Leguminosae, Dipteryxeae). [doctoral thesis] [Campinas, SP]: University of Campinas.

30 Balestra, C. L., Soares, M. P., and Reys, P. (2014). Reproductive biology and pollination of two species of Byrsonima Kunth in a Cerrado fragment in Central Brazil. Revista Biociências 20, 71–81.

31 Amorim, M., and De Marco, P. (2011). Pollination of Byrsonima coccolobifolia: short-distance isolation and possible causes for low fruit production. Brazilian J. Biol. 71, 709–717. doi:10.1590/s1519-69842011000400016.

32 Wnson, S. B., Williams, H. J., Frankie, G. W., and Shrum G. (2006). Floral lipid chemistry of Byrsonima crassifolia (Malpigheaceae) and use of floral lipids by Centris Bees (Hymenoptera: Apidae). Biotropica 29, 76-83.

33 Silva, C. I., Bordon, N. G., Filho, L. C. da R., and Garófalo, C. A. (2012). The importance of plant diversity in maintaining the pollinator bee, Eulaema nigrita (Hymenoptera: Apidae) in sweet passion fruit fields. Rev. Biol. Trop. 60, 1553–1565. doi:10.15517/rbt.v60i4.2073.

34 Reginato, M., and Michelangeli, F. A. (2016). Diversity and constraints in the floral morphological evolution of Leandra s.str. (Melastomataceae). Ann. Bot. 118, 445–458. doi:10.1093/aob/mcw116.

35 Goldenberg, R., and Shepherd, G. J. (1998). Studies on the reproductive biology of Melastomataceae in “cerrado” vegetation. Plant Syst. Evol. 211, 13–29. doi:10.1007/BF00984909.

36 Rodrigues, S. S., Fidalgo, A. O., and Barbedo, C. J. (2017). Reproductive biology and production of seeds and seedlings of Campomanesia pubescens (DC.) O. Berg. J. Seed Sci. 39, 272–279. doi:10.1590/2317-1545v39n3174807.

37 Silva, A. L. G., and Pinheiro, M. C. B. (2009). Reproductive success of four species of Eugenia L. (Myrtaceae). Acta Bot. Brasilica 23, 526–534. doi:10.1590/s0102-33062009000200024.

38 Pires, M. M. Y., and Souza, L. A. (2011). Morphoanatomy and aspects of floral biology from Myrcia guianensis (Aubletet) A. P. de Candolle and Myrcia laruotteana Cambesse (Myrtaceae). Acta Sci. - Biol. Sci. 33, 325–331. doi:10.4025/actascibiolsci.v33i3.6647.

39 Fidalgo, A. O., and Kleinert, A. M. P.(2009). Reproductive biology of six Brazilian Myrtaceae: Is there a syndrome associated with buzz-pollination? New Zeal. J. Bot. 47, 355–365. doi:10.1080/0028825x.2009.9672712.

40 Oliveira, P. E., and Gibbs, P. E. (2000). Reproductive biology of woody plants in a cerrado community of Central Brazil. Flora 195, 311–329. doi:10.1016/S0367-2530(17)30990-8.

41 Montesinos, D., and Oliveira, P. (2014). Reproductive ecology of buzz-pollinated Ouratea spectabilis trees (Ochnaceae) in Brazilian Cerrados. Web Ecol. 14, 79–84. doi:10.5194/we-14-79-2014.

42 Jacobi, C. M., and Carmo, F. F. do (2011). Life-forms, pollination and seed dispersal syndromes in plant communities on ironstone outcrops, SE Brazil. Acta Bot. Brasilica 25, 395–412. doi:10.1590/s0102-33062011000200016.

43 Ospina-Calderón, N. H., Duque-Buitrago, C. A., Tremblay, R. L., and Otero, J. T. (2015). Pollination ecology of Rodriguezia Granadensis (Orchidaceae). Lankesteriana 15, 129–139. doi:10.15517/lank.v15i2.20745.

44 Bernhardt, P. (1990). Pollination ecology of Oxalis violacea (Oxalidaceae) following a controlled grass fire. Plant Systematics and Evolution 128.

45 Albuquerque, A. A. E., Lima H. A., Gonçalves-Esteves V. et. al. (2012). Myrsine parvifolia (Primulaceae) in sandy coastal plains marginal to Atlantic rainforest - a case of anemophily or ambophily? Brazilian Journal of Botany.

46 Amorim, F. W., and Oliveira, P. E. (2006). Estrutura sexual e ecologia reprodutiva de Amaioua guianensis Aubl. (Rubiaceae), uma espécie dióica de formações florestais de cerrado. Rev. Bras. Bot. 29, 353–362. doi:10.1590/S0100-84042006000300003.

47 Alves, T. T. L., Mascena, V. M., Silva, J. N., and Freitas, B. M. (2010). Diversity of insects and frequency of bee visitors in Serjania lethalis in the Chapada do Araripe. Revista Verde 112–116.

48 Forni-Martins, E. R., Marques, M. C. M., and Lemes, M. R. (1998). Floral biology and reproduction of Solanum paniculatum L. (Solanaceae) in the state of São Paulo, Brazil. Rev. Bras. Bot. 21.

49 Tavares, P. R. A., Alves, V. V., Morais, G. A., Polatto, L. P., and Dutra, J. C. S. (2018). Pollen Availability and Behavior of Visiting Bees of Solanum lycocarpum A. St. Hill (Solanaceae). Entomol. News 127, 375–385. doi:10.3157/021.127.0410.

50 Maruyama, P. K., Custódio, L. N., and Oliveira, P. E. (2012). When hummingbirds are the thieves: visitation effect on the reproduction of Neotropical snowbell Styrax ferrugineus Nees & Mart (Styracaceae). Acta Bot. Brasilica 26, 58–64. doi:10.1590/s0102-33062012000100007.

51 Barrows, E. (1976). Nectar Robbing and Pollination of Lantana camara (Verbenaceae). Biotropica 8(2), 132-135. doi:10.2307/2989633

52 Oliveira, P. E. (1997). Reproductive Biology, Evolution and Taxonomy of the Vochysiaceae in Central Brazil. Royal Botanic Gardens Kew.

53 Santos, M. L., Afonso, A. P., and Oliveira, P. E. (1997). Biologia floral de Vochysia cinnamomea Pohl (Vochysiaceae) em cerrados do Triângulo Mineiro, MG. Rev. Bras. Botânica 20, 127–132. doi:10.1590/s0100-84041997000200003.

54 Oliveira, P., and Gibbs, P. (1994). Pollination biology and breeding systems of six Vochysia species (Vochysiaceae) in Central Brazil. J. Trop. Ecol. 10, 509–522. doi:10.1017/S026646740000818X.

55 Mesquita-Neto, J. N., Borges, J. P. R., Sá, T. F. F., Oliveira Teixeira, T. P., Ferreira, I. N. M., Furtado, M. T. R., et al. (2018). Pollen flow and pollinator sharing among synchronopatric species of Psychotria (Rubiaceae). Plant Syst. Evol. 304, 943–953. doi:10.1007/s00606-018-1527-y.

56 Ortiz, P. L., Arista, M., Oliveira, P. E., Talavera, S. (2003). Pattern of flower and fruit
production in Stryphnodendron adstringens, an andromonoecious legume tree of central
Brazil. Plant Biology 5, 592-599.

57 Ishara, K. L., and Maimoni-Rodella, R. de C. S. (2011). Pollination and dispersal systems in a cerrado remnant (Brazilian savanna) in southeastern Brazil. Brazilian Arch. Biol. Technol. 54, 629–642. doi:10.1590/S1516-89132011000300025.

58 Gama, L. U., Barbosa, A. A. A., and de Oliveira, P. E. A. M. (2011). Sistema sexual e biologia floral de Pouteria ramiflora e P. torta (Sapotaceae). Rev. Bras. Bot. 34, 375–387. doi:10.1590/S0100-84042011000300011.

59 Weiblen, G. D. (2002). How to be a fig wasp. Annu. Rev. Entomol. 299-330.

60 Yamamoto, L.F., Kinoshita, L.S., and Martins, F.R. (2007). Síndromes de polinização e de dispersão em fragmentos da Floresta Estacional Semidecídua Montana, SP, Brasil. Acta Botanica Brasilica 21, 553-573.

61 Martins, F. Q., and Batalha, M. A. (2007). Vertical and horizontal distribution of pollination systems in cerrado fragments of Central Brazil. *Brazilian Arch. Biol. Technol.* 50, 503–514. doi:10.1590/S1516-89132007000300016.

62 Lenzi, M., Orth, A. I., and Guerra, T. M. (2005). Ecologia da polinização de Momordica charantia L. (Cucurbitaceae), em Florianópolis, SC, Brasil. *Rev. Bras. Botânica* 28. doi:10.1590/s0100-84042005000300008.

63 Gribel, R., and Hay, J. (1993). Pollination ecology of Caryocar brasiliense (Caryocaraceae) in Central Brazil cerrado vegetation. Journal of Tropical Ecology 9(2), 199-211. doi:10.1017/S0266467400007173

64 Morellato L.P C. Personal observation.

65 Stahl, J. M., Nepi, M., Galetto, L., Guimarães, E., and MacHado, S. R. (2012). Functional aspects of floral nectar secretion of Ananas ananassoides, an ornithophilous bromeliad from the Brazilian savanna. Ann. Bot. 109, 1243–1252. doi:10.1093/aob/mcs053.
