# Supplementary material for: Flowering Phenology and the Influence of Seasonality in Flower Conspicuousness for Bees
Source: Front Plant Sci. 2021 Feb 16;11:594538. doi: 10.3389/fpls.2020.594538 (PMC7921784; doi:10.3389/fpls.2020.594538)
Supplement: Supplementary file 5 [file Table_1.docx]

Supplementary Material

# Supplementary Figures and Tables

## Supplementary Tables

**Table S1.** Cerrado species surveyed and the correspondent flower color, pollinator, human color, bee-color, the presence (P) or absence (N) of the reflectance data, the presence (P) or absence (N) of flowering phenology data, the month of flowering peak and the pollinators references. Bee = bee, col = coleoptera, mot = moth, hum = hummingbird, fly = fly, but = butterfly, smi = small insects, bat = bat, was = wasp, sph = sphingidae. * = pollinator classification based on plant genera. ** = Phenology Lab. personal observation.

| Family | Species | Pollinator | Human color | Bee color | Spectrum | Phenology | Flowering peak | References |
| --- | --- | --- | --- | --- | --- | --- | --- | --- |
| Amaranthaceae | *Pfaffia sp.* | bee | white | bluegreen | P | N | - | 1* |
| Annonaceae | *Duguetia furfuracea* | col | pink | bluegreen | P | P | March | 6 |
| Annonaceae | *Xylopia aromatica* | col | white | bluegreen | P | P | November | 6 |
| Apocynaceae | *Aspidosperma tomentosum* | mot/bee | white | green | P | P | August | 2 |
| Apocynaceae | *Mandevilla pohliana* | mot/bee | pink | blue | P | N | - | 3 |
| Apocynaceae | *Prestonia coalita* | bee | yellow | green | P | N | - | 4 |
| Apocynaceae | *Schubertia grandiflora* | mot/bee | white | bluegreen | P | N | - | 5 |
| Apocynaceae | *Temnadenia violacea* | hum | pink | uvblue | P | N | - | 6 |
| Araliaceae | *Schefflera vinosa* | bee | whitish | green | P | P | July | 6 |
| Araliaceae | *Syagrus flexuosa* | bee | yellow | - | N | P | January | 6 |
| Aristolochiaceae | *Aristolochia esperanzae* | fly | red | bluegreen | P | N | - | 66* |
| Asteraceae | *Baccharis dracunculifolia* | bee | whitish | bluegreen | P | N | - | 6 |
| Asteraceae | *Bidens gardneri* | bee | red | green | P | N | - | 6 |
| Asteraceae | *Chresta sphaerocephala* | bee | pink | blue | P | N | - | 7 |
| Asteraceae | *Chromolaena laevigata* | bee | pink | - | N | P | March | 8 |
| Asteraceae | *Chromolaena sp.* | bee | pink | - | N | P | March | 8 |
| Asteraceae | *Lessingianthu sgrandiflorus* | bee | pink | uvblue | P | N | - | 9* |
| Asteraceae | *Moquiniastrum barrosoae* | bee | whitish | - | N | P | August | 6 |
| Asteraceae | *Moquiniastrum pulchrum* | bee | whitish | - | N | P | May | 10** |
| Asteraceae | *Piptocarpha rotundifolia* | but/bee | whitish | bluegreen | P | P | February | 6 |
| Asteraceae | *Vernonanthura ferruginea* | bee | white | bluegreen | P | N | - | 8* |
| Asteraceae | *Vernonia rubrimea* | bee | whitish | bluegreen | P | N | - | 7,9* |
| Asteraceae | *Vernonia sp. 1* | bee | pink | bluegreen | P | N | - | 7,9,11* |
| Bignoniaceae | *Adenocalymm aaxillare* | bee | yellow | uvgreen | P | P | December | 7 |
| Bignoniaceae | *Amphilophium elongatum* | bee | white | green | P | P | August | 7,12 |
| Bignoniaceae | *Cuspidaria pulchra* | bee | pink | blue | P | N | - | 13* |
| Bignoniaceae | *Fridericia platyphylla* | bee | pink | - | N | P | February | 14 |
| Bignoniaceae | *Fridericia speciosa* | hum | whitish | bluegreen | P | N | - | 7 |
| Bignoniaceae | *Handroanthus ochraceus* | bee | yellow | - | N | P | July | 15 |
| Bignoniaceae | *Jacaranda caroba* | bee | pink | uvblue | P | P | September | 6 |
| Bignoniaceae | *Jacaranda decurrens* | bee | pink | blue | P | N | - | 16 |
| Bignoniaceae | *Jacaranda rufa* | bee | pink | - | N | P | November | 6 |
| Bignoniaceae | *Pyrostegia venusta* | hum | orange | green | P | N | - | 6 |
| Bignoniaceae | *Tabebuia aurea* | bee | yellow | - | N | P | July | 17 |
| Bromeliaceae | *Ananas ananassoides* | hum | pink | blue | P | N | - | 65 |
| Burseraceae | *Protium heptaphyllum* | smi | whitish | - | N | P | December | 64** |
| Calophyllaceae | *Kielmeyera grandiflora* | bee | white | - | N | P | December | 7,18* |
| Caryocaraceae | *Caryocar brasiliense* | bat | whitish | bluegreen | P | P | October | 63 |
| Celastraceae | *Peritassa campestris* | bee | whitish | - | N | P | August | 19* |
| Celastraceae | *Plenckia populnea* | bee | green | green | P | N | - | 6 |
| Connaraceae | *Connarus suberosus* | bee | whitish | bluegreen | P | P | September | 6 |
| Connaraceae | *Rourea induta* | bee | white | bluegreen | P | P | September | 6 |
| Cucurbitaceae | *Momordica charantia* | col | yellow | uvgreen | P | N | - | 62 |
| Dilleniaceae | *Davilla elliptica* | bee | yellow | uvgreen | P | P | April | 20 |
| Ebenaceae | *Diospyros lasiocalyx* | mot | green | - | N | P | September | 6 |
| Erythroxylaceae | *Erythroxylum cuneifolium* | was/bee | whitish | - | N | P | September | 6 |
| Erythroxylaceae | *Erythroxylum suberosum* | was/bee | whitish | green | P | P | September | 21 |
| Erythroxylaceae | *Erythroxylum tortuosum* | was/bee | whitish | - | N | P | September | 21 |
| Fabaceae | *Anadenanthera peregrina var. falcata* | bee | white | bluegreen | P | P | September | 6 |
| Fabaceae | *Ancistrotropis peduncularis* | bee | pink | bluegreen | P | N | - | 6* |
| Fabaceae | *Bauhinia rufa* | bat | white | uvblue | P | P | December | 8 |
| Fabaceae | *Bowdichia virgilioides* | bee | pink | - | N | P | August | 7 |
| Fabaceae | *Chamaecrista desvauxii* | bee | yellow | uvgreen | P | N | - | 22 |
| Fabaceae | *Chamaecrista flexuosa* | bee | yellow | green | P | N | - | 22 |
| Fabaceae | *Chamaecrista ramosa* | bee | yellow | uvgreen | P | N | - | 23 |
| Fabaceae | *Chamaecrista sp.* | bee | yellow | uvgreen | P | N | - | 22 |
| Fabaceae | *Copaifera langsdorffii* | bee | white | - | N | P | November | 24 |
| Fabaceae | *Dalbergia miscolobium* | bee | red | uvblue | P | P | January | 25 |
| Fabaceae | *Dimorphandra mollis* | bee | whitish | - | N | P | December | 26 |
| Fabaceae | *Leptolobium dasycarpum* | bee | white | bluegreen | P | P | November | 22* |
| Fabaceae | *Machaerium acutifolium* | bee | white | bluegreen | P | P | November | 7 |
| Fabaceae | *Machaerium brasiliense* | bee | white | - | N | P | November | 27* |
| Fabaceae | *Mimosa cf. alleniana* | bee | pink | blue | P | N | - | 28 |
| Fabaceae | *Mimosa debilis* | bee | pink | bluegreen | P | N | - | 28* |
| Fabaceae | *Pterodon pubescens* | bee | white | bluegreen | P | P | September | 29 |
| Fabaceae | *Senna rugosa* | bee | yellow | uvgreen | P | P | March | 22 |
| Fabaceae | *Stryphnodendron adstringens* | bee | whitish | green | P | P | October | 56 |
| Fabaceae | *Stryphnodendron rotundifolium* | bee | whitish | bluegreen | P | P | December | 7* |
| Fabaceae | *Tachigali pilgeriana* | bee | pink | blue | P | N | - | 6,7* |
| Iridaceae | *Trimezia juncifolia* | bee | yellow | uvgreen | P | N | - | 64** |
| Lamiaceae | *Hyptidendron sp.1* | bee | pink | blue | P | N | - | 6* |
| Lauraceae | *Ocotea corymbosa* | smi | whitish | - | N | P | September | 61 |
| Lauraceae | *Ocotea pulchella* | fly/bee | whitish | bluegreen | P | P | November | 7 |
| Loganiaceae | *Strychnos pseudoquina* | mot | white | - | N | P | February | 2 |
| Malpighiaceae | *Banisteriopsis campestris* | bee | pink | bluegreen | P | P | March | 6 |
| Malpighiaceae | *Banisteriopsis stellaris* | bee | white | bluegreen | P | P | March | 6 |
| Malpighiaceae | *Byrsonima basiloba* | bee | yellow | green | P | P | September | 30 |
| Malpighiaceae | *Byrsonima coccolobifolia* | bee | pink | bluegreen | P | P | November | 31 |
| Malpighiaceae | *Byrsonima crassifolia* | bee | yellow | green | P | P | September | 32 |
| Malpighiaceae | *Byrsonima intermedia* | bee | yellow | green | P | P | November | 30 |
| Malpighiaceae | *Byrsonima verbascifolia* | bee | yellow | green | P | P | September | 33 |
| Malpighiaceae | *Heteropterys byrsonimifolia* | bee | yellow | uvgreen | P | N | - | 30* |
| Malpighiaceae | *Malpighiaceae sp.1* | bee | white | bluegreen | P | N | - | 6* |
| Malpighiaceae | *Malpighiaceae sp.2* | bee | white | bluegreen | P | N | - | 6* |
| Malvaceae | *Eriotheca gracilipes* | bee | white | bluegreen | P | P | July | 7 |
| Melastomataceae | *Leandra solenifera* | bee | pink | - | N | P | September | 34* |
| Melastomataceae | *Miconia albicans* | bee | white | bluegreen | P | P | September | 6 |
| Melastomataceae | *Miconia ligustroides* | bee | white | bluegreen | P | P | August | 35 |
| Melastomataceae | *Miconia rubiginosa* | bee | white | bluegreen | P | P | November | 35 |
| Melastomataceae | *Miconia stenostachya* | bee | white | - | N | P | September | 35* |
| Siparunaceae | *Siparuna guianensis* | fly | green | - | N | P | October | 60* |
| Moraceae | *Ficus citrifolia* | was | green | - | N | P | September | 59 |
| Myristicaceae | *Virola sebifera* | bee | yellow | - | N | P | March | 7 |
| Myrtaceae | *Campomanesia pubescens* | bee | white | bluegreen | P | P | September | 36 |
| Myrtaceae | *Eugenia bimarginata* | bee | white | bluegreen | P | P | November | 6 |
| Myrtaceae | *Eugenia punicifolia* | bee | white | - | N | P | September | 37 |
| Myrtaceae | *Eugenia pyriformis* | bee | white | - | N | P | October | 6 |
| Myrtaceae | *Eugenia sp.1* | bee | white | - | N | P | November | 6* |
| Myrtaceae | *Myrcia bella* | bee | white | bluegreen | P | P | October | 6 |
| Myrtaceae | *Myrcia guianensis* | bee | white | bluegreen | P | P | September | 38 |
| Myrtaceae | *Myrcia splendens* | bee | white | bluegreen | P | P | October | 39 |
| Myrtaceae | *Psidium grandifolium* | bee | white | - | N | P | December | 6 |
| not. Determined | *not det.sp.1* | bee | pink | blue | P | N | - | 6* |
| not. Determined | *not det.sp.2* | bee | whitish | bluegreen | P | N | - | 6* |
| not. Determined | *not det.sp.3* | bee | white | bluegreen | P | N | - | 6* |
| Nyctaginaceae | *Guapira noxia* | smi/bee | green | - | N | P | September | 40 |
| Nyctaginaceae | *Guapira opposita* | smi/bee | green | green | P | P | September | 6* |
| Ochnaceae | *Ouratea spectabilis* | bee | yellow | uvgreen | P | P | August | 41 |
| Orchidaceae | *Galeandra montana* | bee | pink | uvblue | P | N | - | 42* |
| Orchidaceae | *Rodriguezia sp.* | bee | pink | bluegreen | P | N | - | 43* |
| Oxalidaceae | *Oxalis hirsutissima* | bee | yellow | uvgreen | P | N | - | 44* |
| Primulaceae | *Myrsine guianensis* | bee | whitish | - | N | P | July | 45 |
| Primulaceae | *Myrsine umbellata* | smi | whitish | - | N | P | June | 45 |
| Rubiaceae | *Alibertia sp.* | bee | white | - | N | P | September | 10** |
| Rubiaceae | *Amaioua guianensis* | mot/bee | white | bluegreen | P | P | September | 46 |
| Rubiaceae | *Cordiera sessilis* | smi/bee | white | - | N | P | July | 8* |
| Rubiaceae | *Manettia sp.* | hum | red | bluegreen | P | N | - | 6* |
| Rubiaceae | *Palicourea rigida* | hum/bee | orange | green | P | P | December | 40 |
| Rubiaceae | *Psychotria hoffmannseggiana* | bee | white | bluegreen | P | N | - | 55* |
| Rubiaceae | *Tocoyena formosa* | sph | whitish | bluegreen | P | P | December | 2 |
| Salicaceae | *Casearia sylvestris* | fly | white | - | N | P | September | 7 |
| Sapindaceae | *Serjania lethalis* | bee | white | bluegreen | P | N | - | 47 |
| Sapotaceae | *Pouteria ramiflora* | smi | whitish | - | N | P | September | 58 |
| Sapotaceae | *Pouteria torta* | smi | whitish | - | N | P | July | 58 |
| Smilacaceae | *Smilax brasiliensis* | fly | green | bluegreen | P | N | - | 6 |
| Solanaceae | *Solanum paniculatum* | bee | pink | blue | P | N | - | 48 |
| Solanaceae | *Solanum lycocarpum* | bee | pink | blue | P | N | - | 49 |
| Solanaceae | *Solanum sp.1* | bee | pink | blue | P | N | - | 6* |
| Solanaceae | *Solanum sp.2* | bee | white | bluegreen | P | N | - | 6* |
| Styracaceae | *Styrax ferrugineus* | bee | white | bluegreen | P | P | June | 50 |
| Verbenaceae | *Aegiphila verticillata* | bee | whitish | bluegreen | P | P | October | 6 |
| Verbenaceae | *Lantana camara* | but/bee | orange | green | P | N | - | 51 |
| Verbenaceae | *Lippia balansae* | bee | white | - | N | P | February | 7* |
| Verbenaceae | *Lippia origanoides* | but | white | bluegreen | P | N | - | 57 |
| Vochysiaceae | *Qualea dichotoma* | bee | white | - | N | P | October | 52 |
| Vochysiaceae | *Qualea grandiflora* | sph | yellow | green | P | P | December | 2 |
| Vochysiaceae | *Qualea multiflora* | bee | white | - | N | P | December | 2 |
| Vochysiaceae | *Qualea parviflora* | bee | pink | blue | P | P | January | 52* |
| Vochysiaceae | *Vochysia cinnamomea* | bee | yellow | uvgreen | P | P | August | 53 |
| Vochysiaceae | *Vochysia tucanorum* | bee | yellow | green | P | P | January | 54 |
